# Toward a plant biotechnological application of phyllosphere bacteria

**DOI:** 10.5511/plantbiotechnology.25.0411a

**Published:** 2025-09-25

**Authors:** Rikako Hirata, Yuga Fujinawa, Akira Mine

**Affiliations:** 1 Laboratory of Plant Pathology, Graduate School of Agriculture, Kyoto University, Kyoto 606-8502, Japan

**Keywords:** microbiota, pathogen resistance, phyllosphere bacteria, plant growth promotion, stress tolerance

## Abstract

The phyllosphere, referring to the leaf-dominated aerial parts of plants, represents a vast yet challenging habitat for plant-associated bacteria. A growing body of evidence indicates that phyllosphere bacteria provide host plants with a variety of beneficial effects, including growth promotion, enhanced stress tolerance, and pathogen resistance, garnering significant attention for their potential in biotechnological applications. However, our understanding of the molecular mechanisms underlying these bacterial functions in plant growth and health remains limited. Enhancing the beneficial effects of phyllosphere bacteria requires a deeper understanding of how they adapt to the harsh leaf environment, characterized by limited and unstable water and nutrient availability as well as host-induced defense responses. Moreover, recent studies are beginning to unravel the complex interplay among host plants and members of leaf bacterial communities, which serves as a key driver of the emergence of bacterial functions in the phyllosphere. In this review, we synthesize both early and recent advancements in our understanding of bacterial functions and adaptations in the phyllosphere at the levels of individual strains and communities and propose future research directions to harness phyllosphere bacteria for plant biotechnological applications.

## Introduction

The growing human population and global climate change are placing significant pressure on agricultural food production. Moreover, while modern agriculture achieves high productivity, it heavily depends on chemical fertilizers and pesticides, which cause soil degradation and environmental destruction. Consequently, there is an urgent need to develop sustainable and environmentally friendly agricultural practices that can maintain high productivity amidst the challenges posed by the changing climate. Increasing evidence indicates that plants provide niches for diverse microorganisms and benefit from these complex symbiotic interactions in terms of growth and health (Trivedi et al. 2020; Vandenkoornhuyse et al. 2015). In this review, we explore promising approaches to sustainable agriculture through biotechnological application of these beneficial plant-microbe interactions with a particular focus on phyllosphere bacteria—prokaryotic microorganisms that colonize the aboveground part of plants, referred to as the phyllosphere.

The phyllosphere primarily comprises leaves that host a community of microorganisms that differs in richness and diversity from those inhabiting the root’s interior (root endosphere) and the soil surrounding the root (rhizosphere) (Dong et al. 2019). The rhizosphere is the narrow zone of soil influenced by root secretion of various exudates, including low-molecular-weight compounds such as sugars, amino acids, organic acids, and secondary metabolites, as well as high-molecular-weight compounds like polysaccharides and proteins (Badri and Vivanco 2009). These exudates play key roles in the attraction and colonization of specific microorganisms (Haichar et al. 2014; Lecomte et al. 2018). In contrast to the relatively stable and nutrient-rich environment in the rhizosphere, the phyllosphere is considered a harsh environment for microorganisms at both micro- and macro-scales (Leveau 2019; Vorholt 2012). At the macroscale, daily and seasonal fluctuations of temperature, humidity, and ultraviolet (UV) radiation create challenging conditions. At the microscale, leaf structures such as waxy layers, veins, trichomes, and natural openings (e.g., stomata and hydathodes) alter water and nutrient availability. Adapting to these environmental conditions is especially important for phyllosphere bacteria during their epiphytic life on the leaf surface. Once accessing the leaf interior, phyllosphere bacteria can benefit from the relatively nutrient- and water-rich environment as well as the protection from environmental stresses. However, they must deal with an array of host defense responses triggered by the plant innate immune system (Nobori et al. 2022; Vorholt 2012). Mechanistic understanding of the ecological success of phyllosphere bacteria in these leaf environments is of paramount importance for their biotechnological utilization but is currently fragmented.

Early research on phyllosphere bacteria focused on the ecological functions of individual strains. For instance, *Pseudomonas syringae* was identified as a causal agent of frost injury to plants due to their ability to promote ice nucleation by limiting the supercooling of leaf moisture (Arny et al. 1976). The discovery of the *ice* gene responsible for the ice nucleation activity and the successful utilization of genetically engineered ice nucleation-deficient bacterial strains for the competitive exclusion of ice nucleation-active ones led to the first intentional environmental release of recombinant microorganisms to control plant frost injury (Hirano and Upper 2000). This was followed by the commercialization of *Pseudomonas fluorescens* strain A506 as a biocontrol agent for frost injury and fire blight of pears caused by *Erwinia amylovora* (Lindow and Leveau 2002). Owing to its lack of the ability to produce indole-3-acetic acid (IAA), *P. fluorescens* strain A506 has also been utilized for the competitive exclusion of epiphytic bacteria that cause pear fruit russet by producing IAA (Lindow et al. 1998). These inventions clearly demonstrate the utility of phyllosphere bacteria for biotechnological applications.

**Figure 1:**
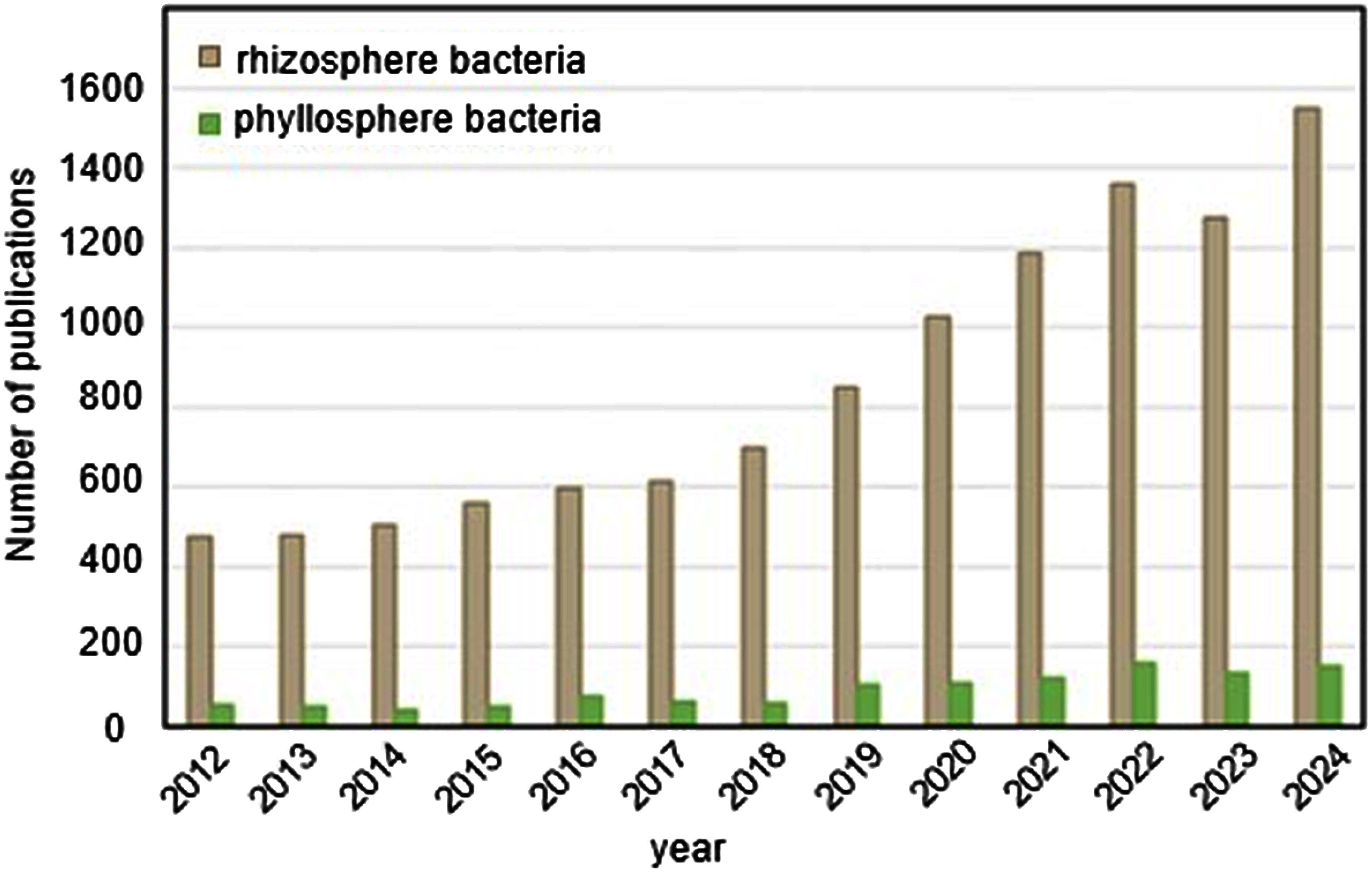
Figure 1. Number of publications retrieved from PubMed using the keywords “rhizosphere bacteria” or “phyllosphere bacteria” from 2012 to 2024.

The total surface area of leaves on Earth is estimated to be twice the land area (Bar-On and Milo 2019), making the phyllosphere a vast habitat for plant-associated bacteria. These bacteria inhabit leaves at an average density of approximately 10^6^–10^7^ bacterial cells per square centimeter (Bulgarelli et al. 2013; Lindow and Brandl 2003). Cultivation-independent analyses powered by next-generation sequencing have uncovered diverse, yet taxonomically defined, communities of phyllosphere bacteria across various host plants (Müller et al. 2016), opening up new avenues for research in this field. Nevertheless, research on phyllosphere bacteria has lagged behind that on rhizosphere bacteria. In 2024, the number of publications retrieved from PubMed using the keyword “phyllosphere bacteria” is less than one-sixth of the rapidly growing number of publications on “rhizosphere bacteria” since the release of the landmark papers on root-inhabiting bacterial communities in 2012 (Bulgarelli et al. 2012; Lundberg et al. 2012) (Figure 1). However, the slight but steady increase in publications on “phyllosphere bacteria” over the past decade (Figure 1) suggests growing interest in this field. Indeed, in analogy to the concept of plant growth-promoting rhizobacterium, the term plant growth-promoting phyllobacterium was recently coined by Rangel and Leveau (2024). Similarly, the term “phylloremediation”, a portmanteau of “phyllosphere” and “bioremediation”, has been introduced to emphasize the potential of phyllosphere bacteria in degrading airborne pollutants as part of environmental remediation strategies (James et al. 2024). Furthermore, the composition of phyllosphere microbial communities has been proposed as an indicator of plant environmental adaptation and stress responses, a concept termed phyllobiomonitoring (Wei et al. 2017). These emerging ideas underscore the increasing recognition of the importance of phyllosphere bacteria in both plant health and environmental sustainability.

Despite the enormous potential for plant biotechnological applications of phyllosphere bacteria, knowledge on the functions of individual bacterial strains remains limited. The genes and mechanisms underlying plant growth-promoting (PGP) traits are still largely unexplored. Furthermore, although stable colonization of phyllosphere bacteria must be key to the emergence of PGP traits, little is known about how individual bacteria adapt to the harsh phyllosphere environment, interact with the host plant, and engage with other members of the microbial community. This review addresses three key aspects crucial for advancing the biotechnological application of phyllosphere bacteria: (1) bacterial functions in the phyllosphere, (2) bacterial adaptation to the phyllosphere environment, and (3) bacterial communities in the phyllosphere. Where appropriate, we compare findings on phyllosphere bacteria with the better-studied rhizosphere bacteria to highlight current challenges and outline future research directions.

## Bacterial functions in the phyllosphere

It has been increasingly recognized that a wide variety of bacteria in the phyllosphere exhibit PGP traits. These PGP traits are mediated by diverse bacterial functions, ranging from production of phytohormones that modulate plant growth and stress tolerance, enhancement of plant nutrient uptake and utilization, to protection against pathogens (He et al. 2024; Singh et al. 2020). This chapter highlights specific examples of these bacterial functions and challenges toward biotechnological applications.

### Bacterial production of phytohormones

Phytohormones are central regulators of plant growth and environmental responses. Among them, auxins, particularly IAA, play a major role in plant growth and organ development (Du et al. 2020). A significant proportion of rhizosphere bacteria are known to synthesize IAA, which has been implicated in bacterial modulation of plant root architecture, nutrient uptake, and tolerance to abiotic stresses (Abedinzadeh et al. 2020; Ahmad Ansari et al. 2023; Amini Hajiabadi et al. 2022; Daraz et al. 2023; Etesami et al. 2015; Kang et al. 2019; Uzma et al. 2022). However, the causal relationship between bacterial IAA production and their PGP effects remains unclear. The production of the growth-promoting phytohormones cytokinin (CK) and gibberellin (GA) is also common for rhizosphere bacteria with PGP traits (He et al. 2024; Keswani et al. 2022). Interestingly, the CK-producing strain of *Azospirillum brasilense* RA-17, but not the non-CK-producing strain RA-18, enhanced wheat growth when colonizing roots (Zaheer et al. 2022), supporting the role of bacterially produced CK in plant growth promotion by rhizosphere bacteria. In contrast, GA production by rhizosphere bacteria appears to be dispensable for PGP effects. For example, *Bradyrhizobium diazoefficiens* produces a bioinactive form of GA, GA_9_, which is then converted to the bioactive GA_4_ in the host plant soybean, resulting in the enhanced bacterial fitness with increased nodule size and bacterial population within nodules, without plant growth promotion (Nett et al. 2022). In the context of phyllosphere bacteria, various species have been characterized as producers of IAA (Alessa et al. 2021; Nutaratat et al. 2017), CKs (Devarajan et al. 2022; Meena et al. 2012), and GAs (Khan et al. 2014). However, little evidence has been provided to support the role of these bacterially produced phytohormones in plant growth promotion. Further molecular and genetic investigation will be necessary to establish a causal link between the PGP effects of both rhizosphere and phyllosphere bacteria and their production of these growth-promoting phytohormones.

The phytohormone ethylene (ET) promotes plant growth at low concentrations, but inhibits growth when present in excess (Pierik et al. 2006). The ET precursor 1-aminocyclopropane-1-carboxylate (ACC) is present in root exudates and acts as a chemical attractant for rhizosphere bacteria (Li T et al. 2019). In the rhizosphere and root endophytic compartments, bacterial ACC deaminases, which metabolize ACC, appear to benefit plants under stress conditions (Orozco-Mosqueda et al. 2020). For example, *Pseudomonas corrugata* CMH3, an ACC deaminase-producing rhizosphere bacterium isolated from saline soils, promoted the growth of barley and oats under salt stress (Chang et al. 2014). Similarly, root endophytic bacteria containing ACC deaminase, such as *P. fluorescens* YsS6 and *Pseudomonas migulae* 8R6, significantly enhanced tomato growth in saline soils, with effects surpassing those of ACC deaminase-deficient mutants (Ali et al. 2014). In the context of phyllosphere bacteria, Herpell et al. (2023) reported that *Paraburkholderia dioscoreae* Msb3 promotes growth of tomato plants when colonizing leaves, although this PGP effect is diminished in ACC deaminase-deficient mutants. These findings highlight that bacterial production of ACC deaminase may play roles in their PGP traits in not only the rhizosphere, but also the phyllosphere, particularly under stress conditions faced by host plants.

The phytohormone abscisic acid (ABA) is a major regulator of plant tolerance to abiotic stresses such as drought, salt, and heavy metals (Vishwakarma et al. 2017). The involvement of plant-derived ABA in modulating plant-microbe interactions is well documented (Mine 2019). Interestingly, the rhizosphere bacterium *Azospirillum brasilense* sp. 245 was shown to increase ABA levels and promote plant growth in an *Arabidopsis thaliana* mutant defective in ABA biosynthesis (Cohen et al. 2015). Furthermore, some ABA-producing rhizosphere bacteria have demonstrated their ability to improve plant stress tolerance in soils contaminated with cadmium or affected by salt (He et al. 2024; Wang et al. 2023; Zhou et al. 2017), although the causal link between bacterial ABA production and stress mitigation in host plants remains to be elucidated. In contrast, very little is known about ABA production by phyllosphere bacteria. Identification and molecular characterization of ABA-producing phyllosphere bacteria will pave the way to explore whether bacterial ABA production in the phyllosphere could contribute to enhanced stress tolerance in plants.

### Bacterial modulation of plant nutrient uptake and utilization

Plants require nitrogen as an essential element but cannot directly utilize atmospheric nitrogen. Bioavailable forms of nitrogen, such as ammonia, are primarily produced through biological nitrogen fixation (BNF), a process carried out by nitrogen-fixing microorganisms. The phyllosphere has emerged as a hotspot for BNF, serving as a key route for nitrogen input into terrestrial ecosystems (Zhu et al. 2023). In maize, BNF by bacterial communities in the mucilage-coated aerial roots and in the xylem have been experimentally demonstrated to contribute to host nitrogen acquisition (Van Deynze et al. 2018; Zhang et al. 2022). Furthermore, 32% of culturable bacteria isolated from maize leaves have been reported to exhibit nitrogen-fixing traits (Abadi et al. 2021). Nitrogen-fixing bacteria typically possess the nitrogenase gene *nifH*, which encodes a critical enzyme for BNF (Gaby and Buckley 2011). A positive correlation between *nifH* gene copy number and nitrogenase activity in leaves (Li Y et al. 2019) further underscores the pivotal role of phyllosphere bacteria in BNF. Importantly, nitrogenase activity was detected in leaves of axenically grown *Jatropha curcas* inoculated with *Methylobacterium* sp., which colonized the leaf surface (Madhaiyan et al. 2015), supporting the occurrence of BNF on leaf surfaces. Although this phyllosphere bacterium promoted host growth and seed set, genetic evidence is lacking to confirm that BNF by this bacterium directly contributes to these PGP effects.

The phyllosphere, particularly the leaf surface, is exposed to oxygen, which dampens nitrogenase activity. However, bacteria have evolved various strategies to create low-oxygen microenvironments. For example, bacterial biofilm formation can decrease interior oxygen levels, maintaining conditions compatible with nitrogenase function (Wang et al. 2017; Wessel et al. 2014). Additionally, bacterial cytochrome *bd* terminal oxidase complexes can rapidly consume oxygen, allowing aerobic bacteria to fix nitrogen (Juty et al. 1997; Kelly et al. 1990). Investigating how nitrogenase activity is maintained in the phyllosphere will be an important research area. In the rhizosphere, the best-studied example of BNF occurs in legume nodules, which are specialized root organs that provide a low-oxygen environment for nitrogen fixation by rhizobia (Zhang et al. 2024). Interestingly, a nitrogen-fixing bacterium, *Cupriavidus taiwanensis* LMG 19424 isolated from *Mimosa* spp., was shown to increase the growth of *A. thaliana* rosette leaves and roots under nitrogen-sufficient conditions. However, a *nifH* knockout strain promoted root growth but failed to enhance rosette leaf growth (Ruiz et al. 2024). While such genetic evidence highlights the role of nitrogen fixation in the rhizosphere, similar studies in the phyllosphere remain lacking. To harness BNF in the phyllosphere as a sustainable alternative to chemical nitrogen fertilizers, future research should focus on elucidating how plants uptake nitrogen produced by phyllosphere bacteria and the extent to which BNF in the phyllosphere contributes to plant growth.

Various rhizosphere bacteria produce siderophores as chelators of iron, an essential micronutrient for plants, thereby facilitating plant iron uptake and promoting growth (Abadi et al. 2020). Siderophore-producing rhizosphere bacteria alleviate iron deficiency in degraded soils, such as saline or drought-affected soils, and support the physiological processes of plants (Singh et al. 2022). In addition, siderophores can sequestrate heavy metal ions from plants, mitigating their toxic effects (He et al. 2024). Interestingly, foliar application of siderophore-producing bacteria increased yields of wheat and soybean, with the degree of yield improvement correlating to the amount of siderophores produced (Sharma et al. 2019). However, a deeper mechanistic understanding is needed to determine whether and how plants uptake irons chelated by bacterial siderophores in the phyllosphere and to quantify the contribution of this nutrient supply to plant growth.

### Bacteria-mediated pathogen resistance in plants

**Figure 2:**
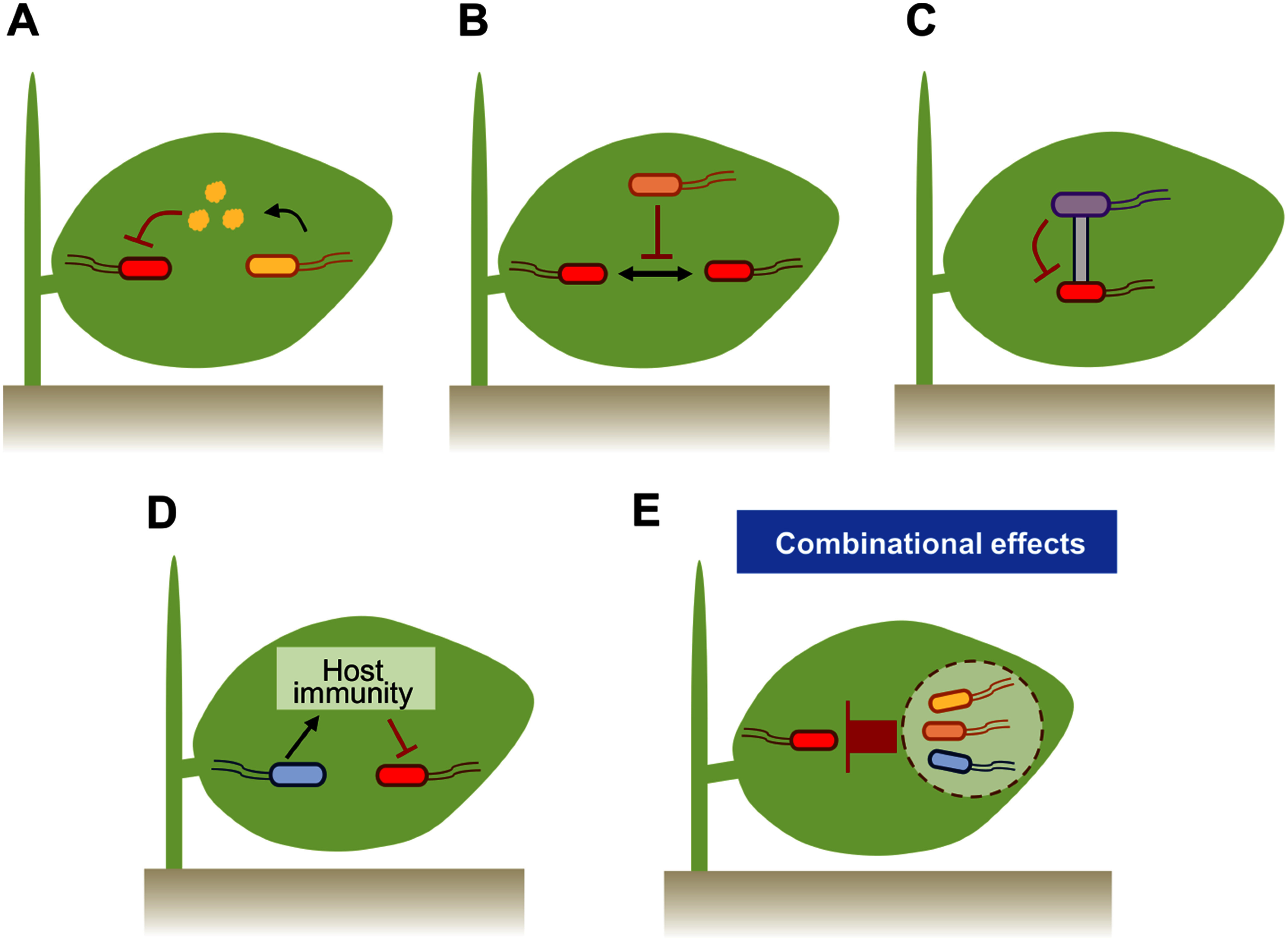
Figure 2. Pathogen suppression by phyllosphere bacteria through various mechanisms: (A) antibiotic production, (B) quorum quenching, (C) T6SS-mediated contact-dependent inhibition, and (D) induction of host immunity. (E) Design of phyllosphere bacterial communities for enhancing their roles in plant protection. T-shaped red bars indicate the suppressive effects of antibiotics (A), quorum quenching (B), T6SS (C), induced host immunity (D), and the collective actions of protective bacterial strains within the community (E) against pathogens.

Pathogens and pests cause significant losses in crop yields globally. While chemical pesticides remain crucial for protecting crops from these threats, their adverse effects on the environment have become a serious concern. In this context, microbe-based biocontrol agents have garnered significant attention and are being increasingly utilized in agriculture (Legein et al. 2020). Here, we focus on living biocontrol bacteria that can suppress pathogens either directly by antagonistic interactions such as antibiotic production (Figure 2A), signal perturbation (Figure 2B), and contact-dependent inhibition (Figure 2C) or indirectly by activating plant disease resistance mechanisms (Figure 2D).

The role of antibiotics in antagonistic microbe-microbe interactions has been well-established, primarily through in vitro binary interaction assays (Legein et al. 2020). This approach was also employed to investigate binary antagonistic interactions between over 200 bacterial isolates from *A. thaliana* leaves (Helfrich et al. 2018). The study revealed that antagonistic activities were predominantly exhibited by a small subset of bacteria, which also showed antagonistic activities against bacterial pathogens *in vitro*. Among these antagonistic bacteria *Brevibacillus* sp. was capable of synthesizing several previously unknown antibiotics, underscoring the potential of phyllosphere bacteria as a source of novel antibiotic compounds. In another study, comparison between wild-type and an antibiotic-negative mutant of a *Brevibacillus brevis* strain demonstrated that the production of the antibiotic, gramicidin S, is required for its biocontrol activity against the fungal pathogen *Botrytis cinerea* on detach leaves (Edwards and Seddon 2001). However, the importance of antibiotic production for pathogen suppression is not always clear. For instance, while the polyketide difficidin produced by *Bacillus amyloliquefaciens* FZB42 effectively inhibited *E. amylovora* *in vitro*, a mutant deficient in difficidin synthesis retained almost full capacity to suppress fire blight symptom in detached apple blossoms (Chen et al. 2009). Further research is needed to firmly establish a functional link between antibiotic production and biocontrol activity in phyllosphere bacteria.

Bacteria use diffusible signaling molecules to engage in chemical cell-to-cell communications, a process known as quorum sensing (QS) (Waters and Bassler 2005). QS is a key virulence mechanism for many phytopathogenic bacteria, regulating population density-dependent infection processes (von Bodman et al. 2003). Perturbation of QS, referred to as quorum quenching (QQ), occurs in virulence regulation and bacterial competition through diverse mechanisms (Grandclément et al. 2016). Interestingly, 14% of bacterial isolates from tobacco leaf surfaces were shown to inhibit QS mediated by N-acyl-homoserine lactones. These QQ phyllosphere bacteria were classified to *Bacillus* sp., *Acinetobacter* sp., *Lysinibacillus* sp., *Serratia* sp., *Pseudomonas* sp., and *Myroides* sp. and were capable of interfering with QS in phytopathogenic bacteria (Ma et al. 2013). Another notable example is *Pseudomonas* spp. strain G, a phyllosphere QQ bacterium capable of rapidly degrading diffusible signal factor, a QS signal used by pathogenic bacteria such as *Xylella fastidiosa* and *Xanthomonas campestris* pv. *campestris*. The QQ activity of this bacterium was required for attenuating disease severity in grape stems inoculated with *X. fastidiosa* and in mustard and cabbage inoculated with *X. campestris* pv. *campestris* (Newman et al. 2008). These findings highlight the potential utility of phyllosphere QQ bacteria as efficient biocontrol agents.

The type VI secretion system (T6SS) plays a crucial role in bacterial competition via intercellular contacts and is found in nearly 25% of Gram-negative bacteria (Ho et al. 2014), including the biocontrol agent *Pseudomonas putida* KT2440 (Bernal et al. 2017). One of the T6SS clusters of *P. putida* KT2440 was found to be essential for its anti-bacterial activity against various phytopathogenic bacteria *in vitro* (Bernal et al. 2017). Notably, the growth of the bacterial pathogen *X. campestris* was inhibited when *P. putida* KT2440 was co-inoculated into *Nicotiana benthamiana* leaves in a T6SS-dependent manner (Bernal et al. 2017), suggesting a prominent role for T6SS in bacterial competition in the phyllosphere. Similarly, Vogel et al. (2021) demonstrated that T6SS is associated with protective *Rhizobium* spp. isolated from *A. thaliana* leaves and that T6SS is indeed required for plant protection by the *Rhizobium* strain Leaf202 against *Pseudomonas syringae* pv. tomato DC3000 (*Pto* DC3000) (Vogel et al. 2021). Interestingly, a recent study revealed an emerging role of T6SS in the anti-fungal activity of the rhizosphere bacteria *Lysobacter enzymogenes* and *P. fluorecens* against *Fusarium graminearum* (Lin et al. 2025). Whether such T6SS-dependent bacteria-fungi interactions occur in the phyllosphere is currently unknown.

Induced systemic resistance (ISR) has been extensively studied as a crucial mechanism by which rhizosphere bacteria prime the whole body of host plants for enhanced defense against pathogens (Pieterse et al. 2014). The critical roles of jasmonic acid (JA) and ET signaling pathways in the establishment of ISR have been well-documented (Pieterse et al. 2014). Interestingly, while colonization of *A. thaliana* roots by *P. fluorescens* SS101 induced ISR against diverse bacterial pathogens and herbivorous pests in leaves, this ISR was independent of JA and ET signaling but dependent on salicylic acid (SA) signaling (van de Mortel et al. 2012). Although the mechanism is less clear compared to rhizosphere bacteria, evidence suggests that certain phyllosphere bacteria protect host plants from pathogens. For instance, *A. thaliana* leaves colonized by the phyllosphere bacterium *Sphingomonas melonis* Fr1 showed heightened expression of defense-related genes and enhanced resistance against *Pto* DC3000 (Innerebner et al. 2011; Vogel et al. 2016). Strikingly, the enhanced resistance induced by *S. melonis* Fr1 was attenuated in *bak1*/*bkk1* mutant plants (Vogel et al. 2016). Since BAK1 and BKK1 function as co-receptors for pattern-recognition receptors (PRRs), which mediate plant innate immunity, (Boutrot and Zipfel 2017), these findings suggest that *S. melonis* Fr1 induces pathogen resistance at least in part through PRR-triggered immunity. Similarly, foliar application of *Bacillus subtilis* DZSY21, isolated from leaves of the medical plant *Eucommia ulmoides* oliv., protected maize leaves from subsequent challenge by the fungal pathogen *Bipolaris maydis* (Ding et al. 2017). This resistance induction was associated with systemic induction of SA and JA marker genes, resembling the process of ISR establishment. Notably, the same effect was also achieved using lipopeptides extracted from this bacterium. In another example, foliar application of the cyclic lipopeptide massetolide A, produced by *P. fluorescens* SS101, induced both local and systemic resistance in tomato leaves against the oomycete pathogen *Phytophthora infestans* (Tran et al. 2007). While accumulating evidence highlights the role of phyllosphere bacteria in conferring local and systemic resistance by producing metabolites that activate plant immune pathways, the molecular identities and mode of actions of these metabolites remain largely uncharacterized.

## Bacterial adaptation to the phyllosphere environment

Stable association with host plants may be important for bacteria to exhibit their PGP traits. However, the phyllosphere presents a challenging environment for bacterial colonization, characterized by limited and unstable availability of water and nutrient, UV radiation, temperature fluctuations, and plant- and microbe-derived antimicrobial compounds. Once inside leaves, phyllosphere bacteria are inevitably recognized by the plant innate immune system, triggering defense responses.

A pioneering study on the phytopathogenic bacterium *Pseudomonas syringae* pv. *syringae* B728a (*Pss* B728a) provided valuable insights into the genomic adaptations enabling survival in the harsh leaf environment. *Pss* B728a is known for its robust epiphytic phase on leaf surfaces, where it establishes and maintains large populations on healthy plants. Comparative genomic analysis of *Pss* B728a and *Pto* DC3000, which does not survive well on leaf surfaces (Boureau et al. 2002), revealed unique genes in *Pss* B728a associated with epiphytic fitness and stress tolerance. These genes are involved in antimicrobial resistance, UV tolerance, ice nucleation, arginine catabolism, and IAA biosynthesis (Feil et al. 2005). A follow-up study on comparative transcriptomics of the epiphytic versus endophytic *Pss* B728a revealed that, during the epiphytic phase on leaf surfaces, genes related to motility, chemotaxis, phosphate uptake, sulfur compound acquisition, and IAA metabolism are upregulated, while, during the endophytic phase in the apoplast, genes associated with metabolism and transport of the nonprotein amino acid GABA as well as phytotoxin biosynthesis are induced (Yu et al. 2013). These findings suggest that *Pss* B728a dynamically shifts its transcriptional profile from acquiring limited nutrients through a search-and-capture strategy on the leaf surface to suppressing host defenses and utilizing the major resources in the apoplast.

In contrast, the responses of non-pathogenic commensal bacteria in the phyllosphere are only beginning to be understood. Nobori et al. (2022) profiled the transcriptome responses of nine phylogenetically diverse commensal bacteria six hours after their infiltration into the apoplast of *A. thaliana* leaves. Unlike the pathogen *Pto* DC3000, which upregulated general metabolic activity and energy production, these commensal bacteria commonly suppressed such cellular activities. The study also revealed that these commensal bacteria triggered plant transcriptional responses associated with PRR-triggered immunity. Similarly, by profiling transcriptome of two leaf endophytic commensal strains one week after infiltration, a recent study by Velásquez et al. (2022) demonstrated that the down-regulation of protein translation and energy production is a shared response between commensal bacteria and a disarmed *Pto* DC3000 in the apoplast. Together, these findings suggest that the induction of plant immune responses may constitute a regulatory mechanism for commensal bacteria to maintain a balanced, healthy relationship with host plants by preventing overgrowth in the apoplast. Although the infiltration and subsequent recovery of large bacterial populations used by these pioneering studies deepened our understanding, new approaches with improved spatial resolution are needed to investigate natural bacterial adaptation processes to the phyllosphere—from initial attachment to a leaf via inoculum sources such as soil, atmosphere, and precipitation, to the establishment of a stable niche within the leaf.

**Figure 3:**
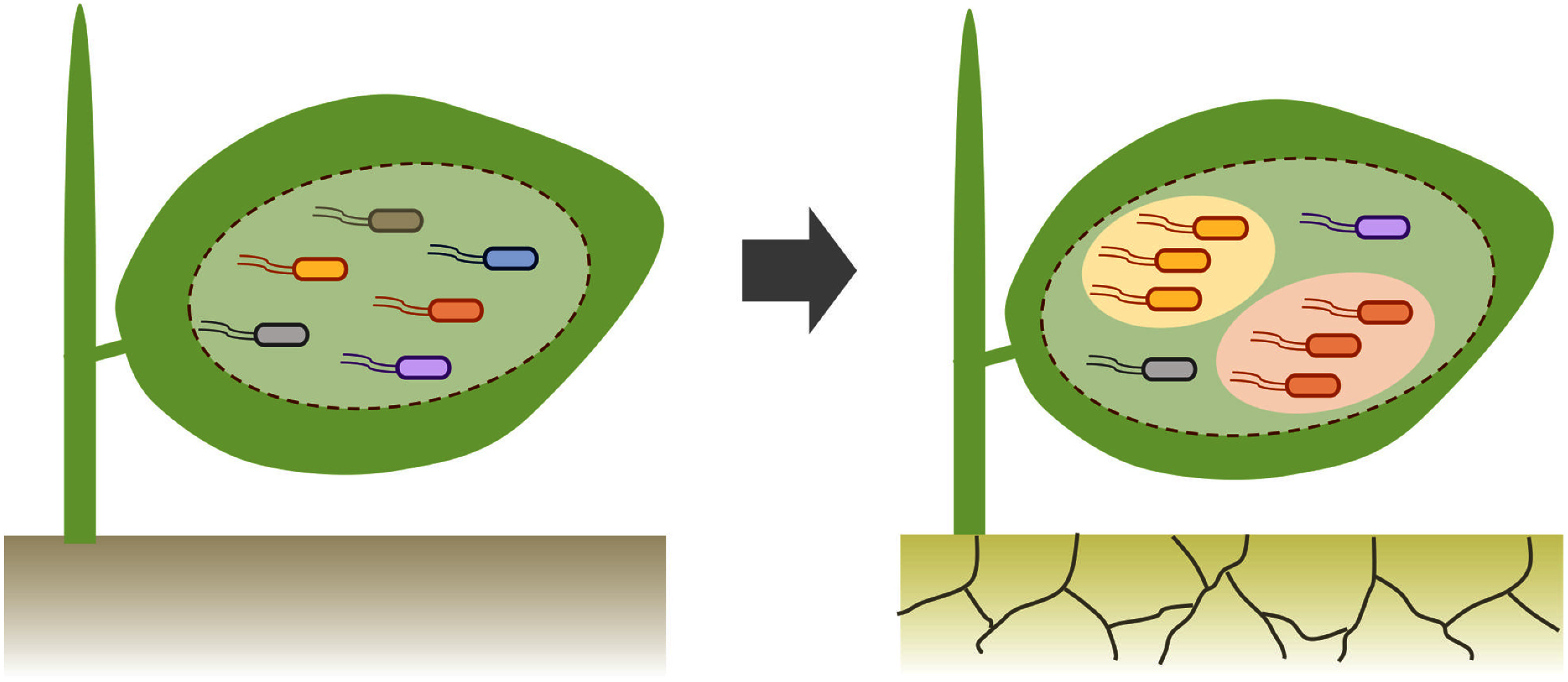
Figure 3. A possible link between shifts in phyllosphere bacterial community and host stress tolerance. Drought, for example, induces significant shifts in the composition of phyllosphere bacterial communities, likely enriching desiccation-tolerant phyllosphere bacteria (represented by the yellow and red bacteria). These desiccation-tolerant bacteria may play a pivotal role in enhancing host tolerance to drought.

The production of biosurfactants is a bacterial strategy to improve leaf wettability, thereby enhancing local water availability and bacterial fitness on leaf surfaces (Bunster et al. 1989; Schreiber et al. 2005). Besides, bacteria form aggregates through the production of extracellular polysaccharides (EPS), which serve as protective agents against desiccation (He et al. 2024; Singh et al. 2020). Such bacteria show significantly higher survival rates on leaves with limited water availability compared to solitary bacteria (Monier and Lindow 2003). Among bacterial strains isolated from the cotton phyllosphere, five strains—*Pseudomonas stutzeri*, *Acinetobacter* spp., *Bacillus mojavensis*, *Pseudomonas chlororaphis*, and *Enterobacter asburiae—*were capable of producing substantial amounts of EPS and thriving on the leaf surfaces of drought-stressed plants. Notably, in addition to its particularly high levels of EPS production, *Acinetobacter* sp., produced ACC deaminase critical for PGP traits. When inoculated onto a drought-sensitive cotton cultivar, this phyllosphere bacterium promoted plant growth under drought conditions (Sharath et al. 2021). Future research should explore the mechanisms underlying the desiccation adaptability of phyllosphere bacteria and its role in their PGP effects under drought stress (Figure 3). In this regard, a recent study revealed a fresh insight into bacterial adaptation to desiccation (Hatfield et al. 2023). The study demonstrated that *Pss* B728a employs a bacteriophytochrome photoreceptor to perceive light as an anticipatory cue, triggering global transcriptional regulation that enhances tolerance to impending water loss. This photo-anticipatory stress tolerance mechanism appears particularly relevant to the epiphytic life of *Pss* B728a, given that water can rapidly evaporate from leaf surfaces upon light exposure at dawn. Whether similar mechanisms operate in nonpathogenic phyllosphere bacteria remains an open question.

Plant-associated bacteria rely on plants for carbon sources, which are particularly scarce in the phyllosphere. Consequently, phyllosphere bacteria have evolved diverse mechanisms to access carbon sources essential for survival in this habitat. For instance, IAA biosynthesis, a trait shared by both pathogenic and mutualistic phyllosphere bacteria, likely contributes to this adaptation, as IAA stimulates cell wall loosening (Majda and Robert 2018), thereby increasing the availability of cell wall-derived saccharides and nutritious molecules diffused from the plant cell to bacteria. In *Sphingomonas* spp., an abundance of TonB receptors, which is involved in the transport of carbohydrate and vitamins, is a notable genetic feature that likely explains their rich abundance in the phyllosphere (Delmotte et al. 2009). Similarly, *Methylobacterium* spp. thrive in this habitat by utilizing single-carbon compounds, such as methanol and methylamine, which are byproducts of plant cell wall metabolism (Palberg et al. 2022). These findings raise an intriguing question from a biotechnological perspective: could manipulating nutrient availability to specific bacteria enhance their colonization efficiency and stability in the phyllosphere? Insights from the rhizosphere may provide clues. A recent study demonstrated that spatially distinct patterns of bacterial colonization along the longitudinal axis of *A. thaliana* roots are regulated by plant SWEET sugar uniporters that affect root metabolite distribution (Loo et al. 2024). Similarly, phyllosphere bacteria also occupy spatially distinct niches on leaf surfaces (Esser et al. 2015; Remus-Emsermann et al. 2014). Exploring plant and bacterial mechanisms driving these niche specializations could be significantly advanced by emerging technologies for spatially resolved, simultaneous mapping of plant and microbial transcriptional responses (Nobori et al. 2025; Saarenpää et al. 2024).

## Bacterial community in the phyllosphere

Although phyllosphere bacteria have traditionally been studied as individual strains, it is increasingly evident that their interactions with host plants are influenced by the co-existing, diverse array of plant-associated microorganisms, collectively called the plant microbiota. Advances in culture-independent analyses, driven by next-generation sequencing, have revealed that phyllospheres across diverse host plants harbor structured bacterial communities dominated by a few major phyla, including Proteobacteria (Pseudomonadota), Actinobacteria (Actinomycetota), Bacteroidetes (Bacteroidota), and Firmicutes (Bacillota) (Müller et al. 2016). Disentangling the complex interactions between plants and their associated bacterial communities is challenging but has become technically feasible with the advent of synthetic communities (SynComs) (Northen et al. 2024; Vorholt et al. 2017)—rationally designed mixtures of representative bacterial strains—and gnotobiotic plant growth systems (Kremer et al. 2021). This chapter highlights recent progress in understanding the intricate interplay between plants and phyllosphere bacterial communities.

### Plant protection by phyllosphere bacteria at the community level

The beneficial effects of rhizosphere bacterial communities include mediating plant protection (Du et al. 2025; Durán et al. 2018) and improving nutrient uptake (Castrillo et al. 2017; Finkel et al. 2020; Wang et al. 2021). In contrast, the beneficial effects of phyllosphere bacterial communities have been predominantly reported in the context of plant protection, although emerging evidence suggests that a soil-derived phyllosphere bacterial community promotes leaf growth in maize (Custódio et al. 2025).

Age-related resistance (ARR) is a widely observed phenomenon, in which older plants exhibit greater pathogen resistance than younger plants (Develey-Rivière and Galiana 2007). While ARR has been traditionally attributed to developmental transitions, Paasch et al. (2023) revealed that *A. thaliana* plants grown axenically in a gnotobiotic system were unable to develop ARR against *Pto* DC3000. Remarkably, ARR was restored upon reintroduction of a SynCom derived from the leaves of healthy *A. thaliana* plants. This microbiota-mediated ARR is reminiscent of the emerging role of mammalian microbiota in the development of host immune system (Zheng et al. 2020).

Another study also employed a SynCom approach to explore community-level functions of phyllosphere bacteria in plant protection against *Pto* DC3000 (Vogel et al. 2021). Using randomly assembled SynComs of ten strains from the culturable *A. thaliana* phyllosphere bacterial community, termed *At*-LSPHERE, the study demonstrated that SynComs frequently showed better protection against *Pto* DC3000 than the individual best-performing protective strains included in the SynComs. Notably, SynCom M10.35 exhibited markedly high protection activity, although each constituent strain individually displayed intermediate or low levels of protection. This enhanced protection required the presence of *Rhodococcus* strain Leaf278; however, the same strain failed to improve protection in another SynCom M10.48, emphasizing the critical role of interactions between community members in host protection. Building on these findings, Emmenegger et al. (2023) trained machine learning models using host protection data of 136 randomly assembled SynComs of five strains, successfully identifying strain combinations that synergistically improved plant protection. These studies provide a framework toward exploring combinatorial functions of bacterial community members to enhance their roles in host protection and beyond (Figure 2E).

The bacterial community of rice panicles has been implicated in the emergence of resistance against the rice false smut fungus *Ustilaginoidea virens* in a susceptible cultivar (Liu et al. 2023). In disease-suppressive rice panicles, certain microbial taxa, including *Lactobacillus* spp., were enriched. Transplantation of such keystone taxa suppressed the expression of a branched-chain amino acid (BCAA) aminotransferase gene in rice, increasing the accumulation of BCAAs in rice panicles. Exposure of *U. virens* to leucine, a BCAA, induced cellular dysfunctions accompanied by apoptosis-like cell death. Thus, the phyllosphere bacterial community prevents rice false smut disease by modulating host amino acid metabolism.

### Mechanisms of bacterial community assembly in the phyllosphere

The assembly of phyllosphere bacterial communities is influenced by both environmental factors and host genetics. For instance, an association study of *A. thaliana* natural populations across the European continent revealed that drought severity and host genetic variations significantly impact leaf bacterial communities (Figure 3) (Karasov et al. 2024). Similarly, the composition of leaf bacterial community was significantly altered under a salt stress condition in controlled laboratory settings (Berens et al. 2019). Host genetic effects can be attributed to plant-derived metabolites, as demonstrated by Unger et al. (2024). This study showed that allyl-glucosinolate produced by the *A. thaliana* ecotype NG2 significantly impacts the bacterial community composition via the enrichment of specific bacterial taxa, mostly Comamonadaceae and Oxalobacteraceae. In contrast, no such effect was observed for 4-methylsulfinylbutyl-glucosinolate, which is produced by the accession Col-0. In rice, a genome-wide association study identified phenylpropanoid 4-hydroxycinnamic acid, a precursor of lignin, as a key metabolite structuring the phyllosphere bacterial community dominated by Pseudomonadales (Su et al. 2024).

**Figure 4:**
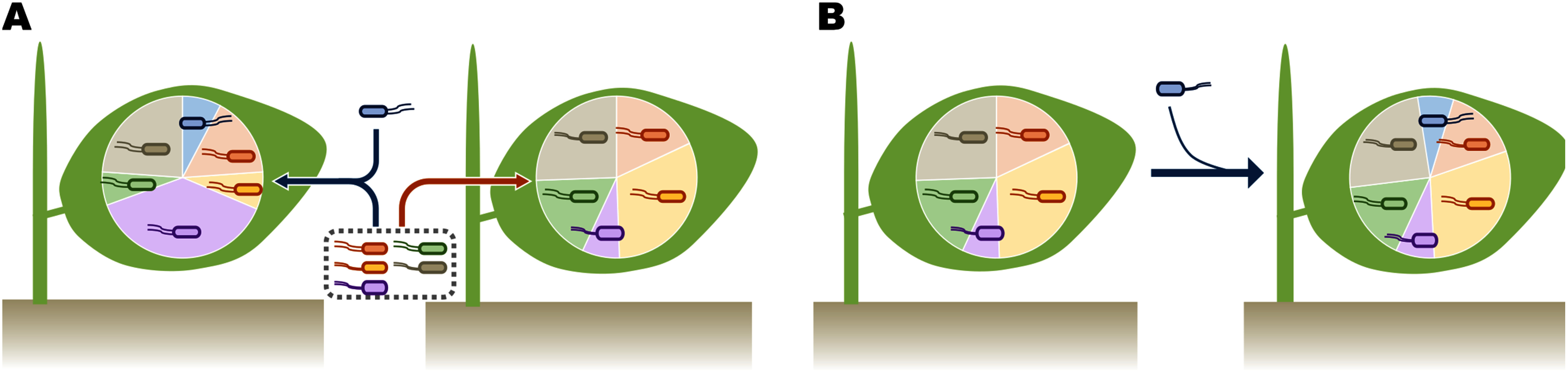
Figure 4. Community-based approaches to enhancing bacterial colonization in the phyllosphere. (A) Keystone taxa (represented by the blue bacterium) greatly alter the composition of phyllosphere bacterial community and may therefore play a crucial role in designing a well-balanced synthetic community. (B) Established bacterial communities are generally stable against late introduction of a new member (represented by the blue bacterium), though the latecomer can often establish a niche, emphasizing the importance of considering the timing and conditions of bacterial inoculation. (A and B) Pie charts depict the relative abundance of community members.

Microbe-microbe interactions also play an important role in shaping leaf bacterial communities. A recent study generated metabolic interaction models using the genome sequences and *in vitro* carbon-source utilization profiles of 224 *At*-LSPHERE strains. According to the carbon source availability of the *A. thaliana* phyllosphere, these models predicted that most interspecies interactions are negative in this niche. Further, experimental validation supported the high accuracy of these predictions, highlighting the critical role of metabolic competition in the assembly of the phyllosphere bacterial community (Schäfer et al. 2023). Another study explored the assembly processes of phyllosphere bacterial communities by conducting drop-out and late introduction experiments with a SynCom of 62 strains from *At*-LSPHERE (Carlström et al. 2019). This approach identified keystone taxa, the removal of which led to substantial shifts in the relative abundances of other strains (Figure 4A). Moreover, the initial community established without the drop-out strains was not significantly altered following the introduction of the drop-out strains, although the drop-out strains could colonize the phyllosphere in most cases (Figure 4B). These findings have practical implications for the biotechnological application of phyllosphere bacteria via foliar administration.

Plant-microbiota interactions can shift to dysfunctional, unbalanced states, known as dysbiosis. In *A. thaliana*, the quadruple mutant *min7 fls2 efr cerk1* (*mfec*), deficient in vesicle-trafficking and PRR-triggered immune signaling showed necrosis and chlorosis under high humidity conditions (Chen et al. 2020). This phenotype was associated with an increased bacterial load and a shift in community composition characterized by an increased proportion of Proteobacteria within the leaves of *mfec* mutant plants, in stark contrast to the Firmicutes-rich community observed in wild-type plants. A SynCom approach provided causal evidence for this dysbiosis; infiltration of a SynCom derived from the leaves of *mfec* plants into axenically grown wild-type plants in a gnotobiotic system reproduced the necrosis, chlorosis, and elevated bacterial load. Similarly, in rice, genetic disruption of 4-hydroxycinnamic acid biosynthesis rendered rice leaves susceptible to the opportunistic pathogen *Xanthomonas* *oryzae* strain TJ1, which is a member of the phyllosphere bacterial community (Su et al. 2024).

Rapid production of reactive oxygen species (ROS) is a hallmark of PRR-triggered immune responses and is dependent on the NADPH oxidase RbohD (Kadota et al. 2015). Recently, ROS has been implicated in preventing dysbiosis. When colonized by a SynCom derived from *At*-LSPHERE, the *A. thaliana* *rbohD* mutant, but not wild-type plants, showed disease symptoms and stunted growth alongside an altered bacterial community (Pfeilmeier et al. 2021). SynCom-based drop-out experiments identified two *Xanthomonas* strains as causal opportunistic pathogens equipped with a type II secretion system (T2SS), which contributed to both disease symptoms and the shift in bacterial community composition (Pfeilmeier et al. 2021, 2024). Interestingly, another study revealed that wild-type *A. thaliana* plants suppress the expression of bacterial T2SS genes through ROS production. This suppression converted the relationship with the *Xanthomonas* strain from dysbiotic to beneficial, thereby enhancing plant resistance to *Pto* DC3000 (Entila et al. 2024).

Together, these findings reveal an emerging picture in which the functionality of the phyllosphere bacterial microbiota is maintained through intricate interactions among plants, bacteria, and their environment (Figures 2D, 3). Disruption of this equilibrium can lead to dysbiosis. A deeper understanding of the mechanisms underlying these tripartite interactions in microbiota homeostasis will be essential for designing bacterial communities that stably exhibit PGP traits under changing environmental conditions.

## Conclusions and perspectives

In the upcoming decades, sustainable and eco-friendly agricultural practices must be developed to meet the demands of a growing global population despite the challenges brought about by climate change. Decades of research have made substantial advances in our understanding of the roles that phyllosphere bacteria play—both as individual strains and as communities—in promoting plant growth and enhancing stress resilience. These findings provide a robust foundation for the biotechnological application of phyllosphere bacteria.

However, many important questions remain unanswered. Is it feasible to genetically engineer phyllosphere bacteria to enhance their PGP traits? How are the PGP trait of individual strains linked to their adaptability to cope with the environmental stresses faced by their host plants? What factors govern the occurrence of dysbiosis in response to shifts in community structures caused by intentional introduction of new members or environmental perturbations? To what extent can the PGP traits of phyllosphere bacteria be enhanced at the community level? And, how transferable are the bacterial functions identified in laboratory studies or in model plants to crops under field conditions? Future research aimed at addressing these questions will be pivotal in advancing the feasibility of using phyllosphere bacteria as a sustainable and eco-friendly alternative to conventional agricultural practices. Such efforts could transform our ability to harness phyllopshere bacteria for improved crop productivity and resilience in the face of a changing climate.

## Abbreviations

ABA: abscisic acid
ACC: 1-aminocyclopropane-1-carboxylate
ARR: age-related resistance
BCAA: branched-chain amino acid
BNF: biological nitrogen fixation
CK: cytokinin
EPS: extracellular polysaccharides
ET: ethylene
GA: gibberellin
IAA: indole-3-acetic acid
ISR: induced systemic resistance
JA: jasmonic acid
PGP: plant growth-promoting
PRR: pattern-recognition receptor
QQ: quorum quenching
QS: quorum sensing
ROS: reactive oxygen species
SA: salicylic acid
SynCom: synthetic community
T2SS: type II secretion system
T6SS: type VI secretion system
UV: ultraviolet
